# Diagnostic Performance of Interleukin-6 (IL-6) and Membrane Glycoprotein Cluster of Differentiation-64 (CD64) for Acute Appendicitis in Girls Presenting with Lower Abdominal Pain

**DOI:** 10.3390/diagnostics16091337

**Published:** 2026-04-29

**Authors:** Eva Filo, Vassileios Mouravas, Dimitrios Sfoungaris, Konstantina Kontopoulou, Asimina Fylaktou, Ioannis Valioulis

**Affiliations:** 1 1st Department of Pediatric Surgery, Aristotle University of Thessaloniki, 54124 Thessaloniki, Greece; vmouravas1@gmail.com (V.M.); surgicalpediatrics@gmail.com (D.S.); 2 Laboratory of Microbiology, “G. Gennimatas” General Hospital, 54635 Thessaloniki, Greece; ntinakont@yahoo.gr; 3 Immunology Department, National Peripheral Histocompatibility Center, Hippocration General Hospital, 11527 Thessaloniki, Greece; fylaktoumina@gmail.com

**Keywords:** appendicitis, interleukin-6 (IL-6), membrane glycoprotein cluster of differentiation-64 (CD64), abdominal pain, biomarkers, ROC, AUC, Alvarado score

## Abstract

**Background:** Acute appendicitis in girls presenting with lower abdominal pain remains a frequent diagnostic dilemma because of the overlap in clinical presentation with gynaecological and non-surgical causes. This study aimed to evaluate the diagnostic performance of IL-6 and CD64 and to compare them with classical inflammatory markers and the Alvarado score. **Methods:** We conducted a prospective observational diagnostic-accuracy study over a three-year period (December 2022 to December 2025) at the First University Paediatric Surgery Clinic, General Hospital of Thessaloniki “Georgios Gennimatas”. Consecutive girls aged ≤16 years presenting with lower abdominal pain were included. The primary outcome was appendicitis (yes/no), defined by the final clinical diagnosis and, where applicable, intraoperative and/or histopathological confirmation. Diagnostic performance was assessed using ROC curves and AUCs with 95% confidence intervals estimated by the DeLong method. The prespecified primary regression model included the Alvarado score and IL-6; IL-6 was summarised on its original scale and log1p-transformed only for regression analyses to account for right-skewness. Additional multivariable models were exploratory. **Results:** Of 74 initially assessed cases, one was excluded (appendiceal neuroendocrine tumour, NET G1), yielding a final sample of 73 girls: 37 with appendicitis and 36 without appendicitis. IL-6 was higher in the appendicitis group (median 19.41 vs. 4.10 pg/mL) and showed moderate discrimination (AUC 0.696). CRP showed lower to borderline performance (AUC 0.595), whereas CD64 did not demonstrate useful discrimination (AUC 0.521). The Alvarado score had the highest discriminatory ability (AUC 0.885). In the subset with complete data, adding IL-6 to the Alvarado score did not materially improve discrimination. **Conclusions:** IL-6 showed moderate diagnostic performance as a standalone biomarker and may be useful as an adjunct, particularly when a clinical score is unavailable or unreliable. CD64 did not add meaningful diagnostic information in this setting. Larger, prespecified studies are required to determine clinically useful cut-offs and to clarify whether IL-6 offers incremental value beyond established clinical assessment.

## 1. Introduction

The term “vermiform appendix” has been traced back to the Egyptian civilization around 3000 BC, when organs were removed during mummification and stored in jars; some jars have been found bearing inscriptions interpreted as referring to an “intestinal worm”. Owing to its apparent predisposition to inflammation, the appendix was long regarded as a vestigial organ [1]. The first recorded anatomical drawing of the appendix is attributed to Leonardo da Vinci in 1508 [2]. In 1886, the American physician Reginald Heber Fitz (1843–1913) introduced the term “appendicitis” in his monograph entitled “Perforating inflammation of the vermiform appendix; with special reference to its early diagnosis and treatment” [1]. Embryologically, development of the appendix begins during the fifth week of gestation and is closely linked to midgut development; by the eighth week of embryonic development, the appendix becomes macroscopically recognizable [1]. In topographic anatomy, the appendix arises from the posterior–medial aspect of the caecum, approximately 1.7 cm from the terminal ileum. Its base lies at the point of convergence of the taeniae coli, and the anterior taenia coli terminates at the base of the appendix [3].

Appendicitis is defined as inflammation of the vermiform appendix. Acute appendicitis is classified as “uncomplicated” or “complicated” by the European Association for Endoscopic Surgery [EAES]. Uncomplicated appendicitis is defined as inflammation in the absence of a phlegmon, free purulent fluid, abscess, or gangrene, whereas complicated appendicitis includes periappendiceal phlegmon with or without a pericaecal abscess, gangrene, or perforation [4]. Peak incidence occurs at 10–14 years in boys and 15–19 years in girls [5]. The lifetime risk of developing acute appendicitis is estimated at 6.7% for women and 8.6% for men, while the lifetime risk of appendicectomy is reported as 23.1% for women and 12% for men [3]. During the acute inflammatory phase, typical histological features include mucosal ulceration, transmural neutrophil infiltration, perforation, and serositis; in chronic inflammation, lymphocytic infiltration predominates [6]. Regarding etiology in the pediatric population, acute appendicitis is most commonly attributed to lymphoid hyperplasia, characterised by excessive proliferation of lymphoid tissue within the appendix, leading to luminal obstruction, inflammation, and localised ischemia [7]. In the context of ongoing inflammation, particularly in the absence of timely intervention, complications may arise, including perforation, periappendiceal abscess, and peritonitis [8]. In such cases, the inflammatory cascade leads to increases in pro-inflammatory cytokines and acute-phase reactants, including interleukin-6 (IL-6), tumour necrosis factor-α (TNF-α), and C-reactive protein (CRP), which have been associated with greater disease severity [9]. In clinical practice, the diagnosis of appendicitis in children relies on the clinical presentation, physical examination findings, and laboratory and imaging assessment [10]. Several scoring systems that combine clinical features with laboratory values are available; the Pediatric Appendicitis Score and the Alvarado score are among the most widely used, with the Alvarado score being the most commonly applied in many settings [7,11]. The Alvarado score comprises migratory pain (1 point), anorexia (1 point), nausea/vomiting (1 point), right lower quadrant tenderness (2 points), rebound tenderness (1 point), temperature > 37.3 °C (1 point), leukocytosis > 10,000/mm^3^ (2 points), and neutrophil left shift > 75% (1 point), for a total of 10 points. Conventional interpretation considers scores of 1–4 as low probability, 5–6 as intermediate probability, and 7–10 as high probability of appendicitis. Although clinically useful, the score is not disease-specific and may be misleading in non-appendiceal causes of acute abdominal pain or systemic inflammatory presentations; in girls, the differential diagnosis further includes ovarian and other adnexal pathology. Imaging therefore remains an important complementary component of assessment in routine practice, most commonly with ultrasonography as the first-line modality; however, real-world performance may vary according to operator experience and local workflow [11,12].

In 1998, the World Health Organization defined a biomarker as “any substance, structure or process that can be measured in the body or its products and influence or predict the incidence of outcome or disease” [13]. Serum biomarkers are objectively measurable and interpretable indicators that reflect physiological and pathological processes, as well as pharmacological responses to therapeutic interventions. They are used to identify, differentiate, and evaluate pathological states, to assess disease severity, and to guide the determination, monitoring, and prognostication of treatment response [14].

In a study published in BMC Surgery in 2006, Ulrich Sack and colleagues reported that white blood cell count, CRP, and IL-6 were directly associated with the severity of appendiceal inflammation in children [15]. Identification of severe appendicitis was supported by IL-6 or CRP, but not by white blood cell count; nonetheless, CRP and IL-6 were considered complementary markers that may assist in identifying the need for prompt surgical management rather than serving as stand-alone diagnostic tests [15]. In a 2022 systematic review published in World Journal of Pediatrics, Arredondo Montero and colleagues concluded that the sensitivity and specificity of serum IL-6 for diagnosing uncomplicated acute appendicitis in the pediatric population are moderate, but appear higher for complicated appendicitis [13]. They also noted an apparent association between serum IL-6 levels and the duration [in hours] of abdominal pain in children with acute appendicitis, and highlighted the need for further studies to evaluate this biomarker in distinguishing complicated from uncomplicated disease [13].

Neutrophil CD64 expression has been investigated in recent years as a biomarker of infection and sepsis. It has features that support clinical applicability: under resting conditions, neutrophil CD64 expression is low, but following activation it increases markedly within a few hours. CD64 has been described as a promising sepsis biomarker, superior to CRP and potentially outperforming procalcitonin. Importantly, neutrophil CD64 appears to perform comparably across age groups, including adults, neonates, and infants [16].

Interleukin-6 [IL-6] was initially described under several names—B-cell stimulatory factor-2 [BSF-2], interferon-β2 [IFN-β2], hybridoma/plasmacytoma growth factor, macrophage–granulocyte inducer type 2, and hepatocyte stimulating factor [HGF]—reflecting its diverse biological activities. Following its cloning in 1986 and the characterization of its functional properties, these activities were attributed to a single cytokine, subsequently termed interleukin-6 [IL-6] [17]. IL-6 plays a central role in inflammation, acting as a key inducer of CRP, fibrinogen, and serum amyloid A, among many other mediators [17]. In a study of 137 children aged <15 years with appendicitis, Elliver and colleagues observed higher serum concentrations of IL-6 and IL-10 and lower concentrations of TNF-β in complicated appendicitis. Elevated serum IL-6 was associated with increased risk of complicated appendicitis, whereas serum IL-1α, IL-1β, IL-2, IL-10, IL-17A, and TNF-β were not similarly associated. The area under the ROC curve [AUC] for IL-6 was 0.75, indicating moderate ability to identify complicated acute appendicitis [18].

## 2. Materials and Methods

The objectives of this study were: (a) to evaluate the diagnostic performance of IL-6 and CD64 in discriminating appendicitis from non-appendicitis among girls presenting with lower abdominal pain; and (b) to assess whether IL-6 provides incremental diagnostic information beyond the Alvarado score and basic laboratory indices (WBC, CRP, and white cell differentials).

### 2.1. Study Design

We conducted a prospective single-centre observational diagnostic-accuracy study over a three-year period (December 2022 to December 2025) at the First University Paediatric Surgery Clinic, General Hospital of Thessaloniki “Georgios Gennimatas”. Consecutive female paediatric patients presenting to the Emergency Department with lower abdominal pain were included, and all underwent routine clinical, laboratory, and imaging assessment according to the treating team’s standard practice.

#### 2.1.1. Study Population and Eligibility Criteria

**Figure 1:**
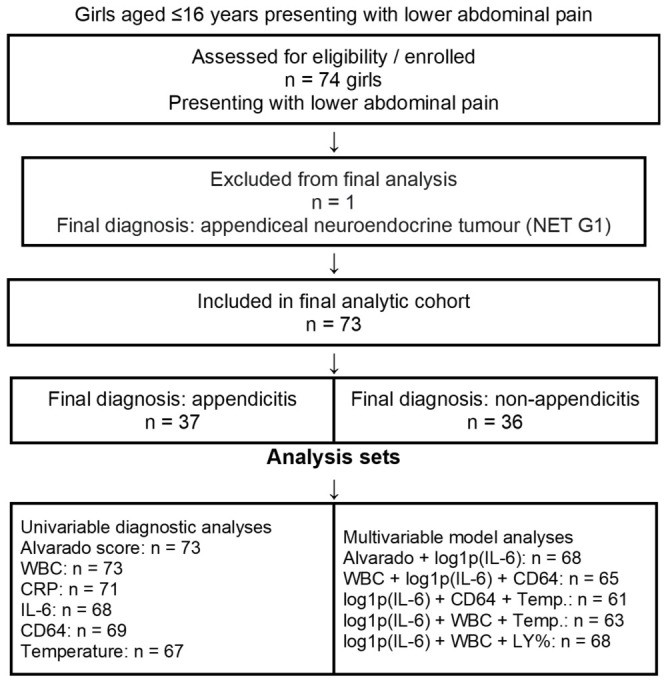
STARD-style participant flow diagram.

Inclusion criteria were: (a) girls aged ≤16 years presenting with lower abdominal pain; (b) absence of major comorbidities; and (c) completion of the initial diagnostic evaluation and follow-up until a final diagnosis was established. Exclusion criteria were: (a) oncology patients; (b) patients with major comorbidities or previous relevant lower abdominal surgery (as specified in the protocol); and (c) cases with an unsuitable blood sample for laboratory processing. In addition, one case operated on for suspected appendicitis but ultimately diagnosed with an appendiceal neuroendocrine tumour (NET G1) was excluded from the final analysis, as prespecified (Figure 1). Imaging formed part of routine clinical care as indicated, but imaging findings were not entered into the analysed biomarker models because acquisition and reporting were not standardised for research use across all participants.

#### 2.1.2. Definition of Groups and Outcome

The primary diagnostic outcome was the presence of appendicitis (yes/no). The reference standard was the final diagnosis established after completion of the clinical work-up and follow-up. In operated appendicitis cases, this diagnosis was supported by intraoperative findings and histopathological examination of the resected appendix. In non-operated and non-appendicitis cases, final classification was based on the documented final clinical diagnosis after completion of the diagnostic work-up and follow-up. The Alvarado score was treated strictly as a comparator variable and was not used to define outcome status. Descriptors such as acute appendicitis, appendiceal empyema, gangrenous appendicitis, or appendiceal plastron were retained only as secondary descriptive labels within the appendicitis group and were not analysed as separate outcome classes.

### 2.2. Variables and Indices

We recorded demographic and anthropometric data (age, weight, height, BMI), clinical signs/measurements (e.g., temperature), and routine laboratory parameters (e.g., WBC, lymphocyte percentage, CRP), as well as the biomarkers of interest: IL-6 (pg/mL) and CD64 (X-mean, arbitrary units). The Alvarado score was calculated according to the standard criteria.

### 2.3. Laboratory Measurements

IL-6 was measured using an immunochemiluminescent method on an automated immunoassay analyzer. CD64 was measured by flow cytometry at a collaborating laboratory (due to lack of availability at the reference hospital). Routine haematology was performed on the UniCel DxH 600 Coulter Cellular Analysis System (Beckman Coulter, Brea, CA, USA). Routine biochemistry was performed on cobas c 501/cobas 6000 analyzers (Roche Diagnostics GmbH, Mannheim, Germany) using the laboratory’s established automated methods, and CRP was measured by turbidimetry on the same platform. IL-6 was measured by electrochemiluminescence immunoassay on the cobas e 411/Elecsys platform (Roche Diagnostics GmbH, Mannheim, Germany). CD64 was measured by flow cytometry at the collaborating laboratory using the Navios EX platform (Beckman Coulter, Brea, CA, USA) at the collaborating laboratory, due to lack of local availability at the reference hospital. For interpretive context, the institutional laboratory reference intervals were as follows: IL-6 < 7 pg/mL; WBC 4.0–11.0 × 10^3^/μL; HCT 36–47%; HGB 12–16 g/dL; neutrophils 40–70%; lymphocytes 20–45%; monocytes 2–9%; platelets 150–400 × 10^3^/μL; and CRP < 0.5 mg/dL. These values were used as local laboratory reference intervals and not as appendicitis-specific diagnostic thresholds.

### 2.4. Sample Size

Sample-size planning was performed a priori at the protocol stage using G*Power software (version 3.1.9.7) for a two-sided Wilcoxon–Mann–Whitney comparison between two independent groups, which reflected the original primary between-group biomarker comparison framework of the study. The calculation assumed a two-sided significance level (α) of 0.05, statistical power of 80%, and a standardized effect size of 1.0, yielding a minimum required total sample of 46 participants. The planned allocation ratio was 1:1.

Because the broader protocol was conducted in a real-world paediatric emergency setting in which some clinically important final diagnoses were relatively uncommon, the final recruitment target also incorporated feasibility considerations in addition to the formal a priori calculation. During the recruitment period, 74 girls were enrolled and 73 were retained in the final analytic cohort after prespecified exclusion of one case with appendiceal neuroendocrine tumour, thus exceeding the minimum sample size derived from the original protocol assumptions.

The present manuscript should therefore be interpreted as an appendicitis-focused diagnostic-accuracy analysis nested within that broader prospective feasibility-based protocol. Accordingly, the sample-size justification applies primarily to the original between-group comparison framework and not to formal development or validation of multivariable clinical prediction models. For this reason, all multivariable regression models in the present study were prespecified as exploratory and are interpreted cautiously.

### 2.5. Statistical Analysis

Continuous variables are reported as mean ± SD when distributions were compatible with parametric approaches, or as median (IQR) when departures from normality were evident. Distributional shape was assessed using visual inspection of histograms and Q–Q plots, together with summary statistics; homoscedasticity was considered when selecting between parametric and non-parametric comparisons. Two-group comparisons used Welch’s *t*-test or the Mann–Whitney U test, as appropriate. Effect sizes are reported as Hedges’ g for mean differences and r for non-parametric comparisons. Diagnostic performance of individual markers and models was evaluated using ROC curves and AUC, with 95% confidence intervals computed by the DeLong method [19].

The prespecified primary model was a logistic regression including the Alvarado score and log1p(IL-6), to address right-skewness in IL-6 and the presence of zero/very low values. Model performance was assessed using AUC in the common subset of observations with complete data for the included terms. Exploratory multivariable approaches without a clinical score were also examined, combining log1p(IL-6) with objective laboratory/clinical indicators (e.g., WBC, lymphocyte percentage, temperature, CD64), and evaluated via AUC. In addition, an exploratory IL-6 threshold (≥7 pg/mL), anchored to the laboratory upper reference limit rather than to a prespecified appendicitis-specific clinical cut-off, was examined, with sensitivity and specificity reported descriptively in the results. Analyses were performed using an available-case approach, with explicit reporting of the sample size (N) for each marker/model (e.g., differing N for IL-6, CD64, CRP, temperature). To assess the influence of extreme values, winsorization at the 99th percentile was applied to IL-6 and CD64, and selected ROC models were re-evaluated; this did not materially change the conclusions.

### 2.6. Software

Statistical analyses were implemented in Python 3.11.2 (main, 28 April 2025, 14:11:48) [GCC 12.2.0] and automated via custom scripts. The following packages were used: pandas 2.2.3 (data management/cleaning), NumPy 1.24.0 (numerical computation), SciPy 1.14.1 (statistical tests/utilities), statsmodels 0.14.3 (logistic regression/Logit and calibration indices), scikit-learn 1.4.2 (ROC/AUC, Brier score), and matplotlib 3.7.5 (figures).

## 3. Results

Over the three-year study period (December 2022 to December 2025) at the First University Paediatric Surgery Clinic, General Hospital of Thessaloniki “Georgios Gennimatas”, 74 girls aged 4.5 to 15.5 years presenting with lower abdominal pain were assessed. One patient who underwent surgery for suspected appendicitis was found on histopathological examination to have an appendiceal neuroendocrine tumour (NET G1) and was excluded, leaving a final analytic sample of 73 girls. The appendicitis group comprised 37 patients and the non-appendicitis group 36 patients. Within the appendicitis group, the operative/final-diagnosis spectrum was described clinically as acute appendicitis (n = 20), appendiceal empyema (n = 7), gangrenous appendicitis (n = 8), and appendiceal plastron (n = 2). The non-appendicitis group was heterogeneous and included mainly ovarian/adnexal pathology, mesenteric lymphadenitis, nonspecific/atypical abdominal pain, and a small number of other diagnoses such as respiratory or urinary infection and one Müllerian anomaly.

### 3.1. Demographic and Anthropometric Characteristics

**Figure 2:**
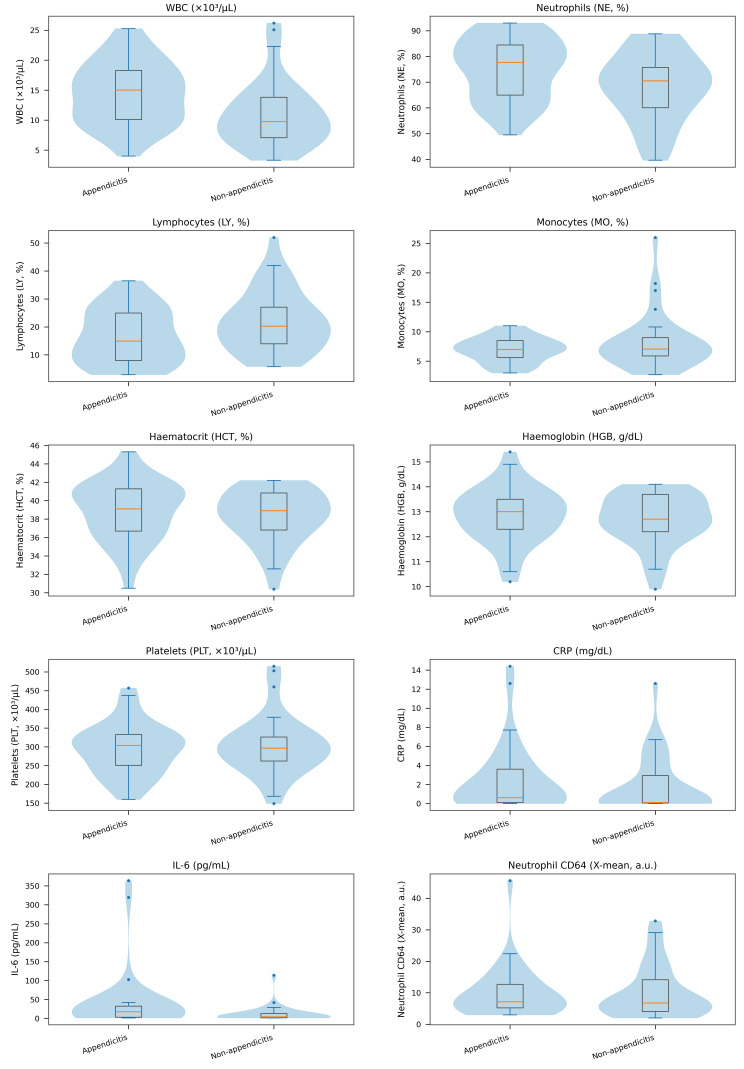
Violin plots with overlaid boxplots of haematological/biochemical parameters and biomarkers at admission by final diagnosis (appendicitis vs. non-appendicitis). Dots indicate outliers (values beyond 1.5 × IQR from the quartiles).

The final analytic sample comprised 73 girls presenting with lower abdominal pain. Of these, 37 (50.7%) were assigned to the appendicitis group and 36 (49.3%) to the non-appendicitis group. The distribution of participants across the two groups was essentially balanced, facilitating interpretation of between-group comparisons. Age was similarly distributed between groups. The median age was 13.0 years (IQR 11.0–14.0) in the appendicitis group and 12.0 years (IQR 9.38–14.0) in the non-appendicitis group, with no statistically significant difference (*p* = 0.424) and a very small effect size (r = −0.109). A similar pattern was observed for anthropometric parameters. Body weight, height, and body mass index (BMI) did not differ significantly between groups (*p* = 0.581, *p* = 0.390, and *p* = 0.168, respectively), and the corresponding effect sizes were small (Hedges’ g = −0.129 for weight, g = 0.200 for height, and g = −0.333 for BMI). Regarding data completeness, BMI was unavailable in four cases; therefore, descriptive analyses for BMI were based on available observations (n = 36 in the appendicitis group and n = 33 in the non-appendicitis group) (Table 1). Overall, the demographic and anthropometric profiles indicate that the two groups were broadly comparable at baseline (Figure 2).

**Table 1:** Demographic and anthropometric characteristics of girls presenting with lower abdominal pain, stratified by final diagnosis (appendicitis vs. non-appendicitis).

Variable	Appendicitis (n = 37)	Non-Appendicitis (n = 36)	*p*-Value	Statistical Test	Effect Size
Age (years), median (IQR)	13.00 (11.00–14.00)	12.00 (9.38–14.00)	0.424	Mann–Whitney U	r = −0.109
Weight (kg), mean ± SD	45.97 ± 13.85	47.88 ± 15.39	0.581	Welch’s *t*-test	g = −0.129
Height (cm), mean ± SD	151.05 ± 14.52	147.97 ± 15.89	0.390	Welch’s *t*-test	g = 0.200
BMI (kg/m^2^), mean ± SD	19.87 ± 4.00 (n = 36)	21.27 ± 4.34 (n = 33)	0.168	Welch’s *t*-test	g = −0.333

Note: Age is reported as median (IQR). Weight/height/BMI are reported as mean ± SD. BMI had missing values in 4 cases.

The clinical characteristics at presentation of girls with lower abdominal pain are shown in Table 2, comparing the appendicitis group (n = 37) with the non-appendicitis group (n = 36).

**Figure 3:**
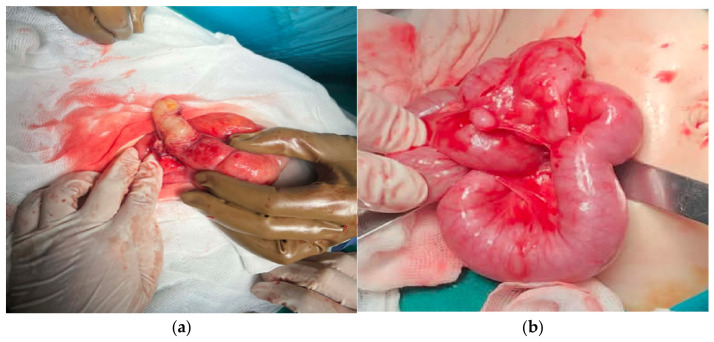
Representative clinical images from the appendicitis (**a**) and non-appendicitis (**b**) groups, provided for illustrative clinical context only. These images were not used as index tests, cohort-defining criteria, or statistical inputs in the present diagnostic-accuracy analysis.

Haematological/biochemical parameters and biomarkers at admission are presented in Table 3. For each variable, descriptive statistics (mean ± SD or median [IQR]) were selected according to the within-group distribution, while between-group comparisons were performed using appropriate tests (Welch’s *t*-test or the Mann–Whitney U test) and are accompanied by effect sizes. The distributions of these markers are illustrated in Figure 2. Figure 3 has representative clinical images from the appendicitis (a) and non-appendicitis (b) groups, provided for illustrative clinical context only.

### 3.2. Exploratory ROC and Multivariable Analyses of IL-6 and CD64

In this cohort of girls presenting with lower abdominal pain (N = 73), IL-6 showed moderate univariable discriminatory ability for distinguishing appendicitis from non-appendicitis (AUC = 0.696, N = 68), outperforming CRP (AUC = 0.595, N = 71) and clearly exceeding CD64, which did not provide clinically useful discrimination in this dataset (AUC = 0.521, N = 69). These results support IL-6 as a potentially informative adjunct biomarker, but not as a stand-alone diagnostic test (Table 4).

**Figure 4:**
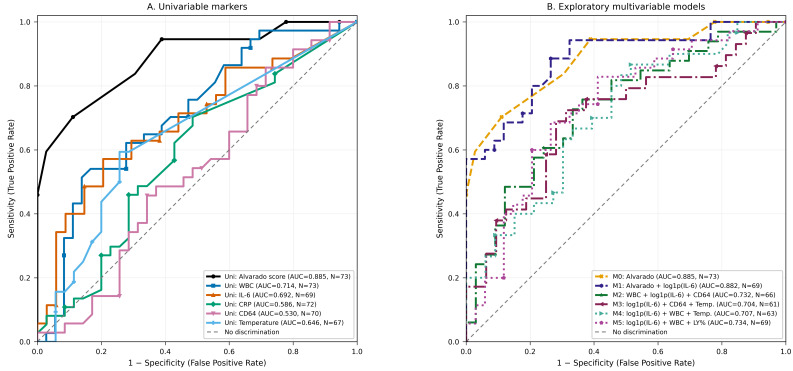
Two-panel receiver operating characteristic (ROC) analysis for the diagnosis of appendicitis. **Panel** **A** shows ROC curves for univariable markers, including the Alvarado score, white blood cell count (WBC), interleukin-6 (IL-6), C-reactive protein (CRP), neutrophil CD64 (X-mean), and temperature. **Panel B** shows ROC curves for exploratory multivariable logistic regression models: M0 (Alvarado), M1 (Alvarado + log1p[IL-6]), M2 (WBC + log1p[IL-6] + CD64), M3 (log1p[IL-6] + CD64 + temperature), M4 (log1p[IL-6] + WBC + temperature), and M5 (log1p[IL-6] + WBC + lymphocyte percentage). The diagonal dashed line indicates no-discrimination performance. Area under the curve (AUC) values and sample sizes (N) are reported in the corresponding panel legends. Ninety-five percent confidence intervals were estimated using the DeLong method. log1p(IL-6) denotes ln(IL-6 + 1).

Within exploratory multivariable models that did not incorporate a clinical score, IL-6 retained a consistent contribution when combined with objective laboratory/clinical indicators, with the best-performing specifications achieving overall moderate discrimination (e.g., log1p(IL-6) + WBC + LY%: AUC = 0.733, N = 68; log1p(IL-6) + WBC + temperature: AUC = 0.697, N = 63; log1p(IL-6) + CD64 + temperature: AUC = 0.692, N = 61). By contrast, when the Alvarado score was available, adding IL-6 did not materially improve the discriminatory performance of the clinical model (AUC = 0.885 for Alvarado alone and AUC = 0.885 for Alvarado + log1p(IL-6) in the common subset). These models should be interpreted cautiously because they were exploratory complete-case analyses in a modest sample and were not developed using penalisation or external validation (Table 5 and Figure 4).

Sensitivity analyses addressing the influence of extreme values (winsorization at the 99^th^ percentile for IL-6 and CD64) did not alter the conclusions, supporting the robustness of the main qualitative findings.

**Table 4:** Univariable diagnostic performance of individual clinical and laboratory markers for discriminating appendicitis from non-appendicitis in girls presenting with lower abdominal pain.

Marker	N	AUC	95% CI (DeLong)
Alvarado score	73	0.885	0.811–0.959
WBC (×10^3^/µL)	73	0.714	0.594–0.834
IL-6 (pg/mL)	68	0.696	0.568–0.823
CRP (mg/dL)	71	0.595	0.461–0.729
CD64 (X-mean, a.u.)	69	0.521	0.382–0.660
Temperature (°C)	67	0.658	0.536–0.780

**Table 5:** Exploratory multivariable logistic regression models for discriminating appendicitis from non-appendicitis in girls presenting with lower abdominal pain.

Predictors	N	AUC (95% CI)	Odds Ratios (OR, 95% CI)	*p*-Values
Alvarado	73	0.885 (0.811–0.959)	Alvarado: 2.279 (1.585–3.277); Intercept: 0.008 (0.001–0.071)	Alvarado: <0.001; Intercept: <0.001
Alvarado + log1p(IL-6)	68	0.885 (0.805–0.964)	Alvarado: 2.244 (1.514–3.326); log1p(IL-6): 1.031 (0.604–1.760); Intercept: 0.008 (0.001–0.079)	Alvarado: <0.001; log1p(IL-6): 0.912; Intercept: <0.001
WBC + log1p(IL-6) + CD64	65	0.732 (0.608–0.856)	WBC: 1.078 (0.971–1.196); log1p(IL-6): 1.767 (1.064–2.935); CD64: 0.963 (0.892–1.039); Intercept: 0.156 (0.033–0.743)	WBC: 0.159; log1p(IL-6): 0.028; CD64: 0.327; Intercept: 0.020
log1p(IL-6) + CD64 + Temperature	61	0.692 (0.555–0.829)	log1p(IL-6): 1.753 (1.020–3.014); CD64: 0.935 (0.857–1.020); Temperature: 2.025 (0.693–5.913); Intercept: 0.000 (0.000–214,907.652)	log1p(IL-6): 0.042; CD64: 0.131; Temperature: 0.197; Intercept: 0.178
log1p(IL-6) + WBC + Temperature	63	0.697 (0.566–0.828)	log1p(IL-6): 1.428 (0.845–2.412); WBC: 1.081 (0.966–1.209); Temperature: 1.388 (0.577–3.341); Intercept: 0.000 (0.000–101,480,781.803)	log1p(IL-6): 0.183; WBC: 0.173; Temperature: 0.464; Intercept: 0.396
log1p(IL-6) + WBC + LY%	68	0.733 (0.610–0.855)	log1p(IL-6): 1.600 (0.987–2.593); WBC: 1.103 (0.977–1.245); LY%: 1.003 (0.939–1.072); Intercept: 0.096 (0.005–1.807)	log1p(IL-6): 0.056; WBC: 0.112; LY%: 0.918; Intercept: 0.118

Notes: Outcome: appendicitis (1) vs. non-appendicitis (0). Each model was analysed on a complete-case basis; therefore, N varies across models. Log1p(IL-6) = ln (IL-6 + 1). AUCs with 95% confidence intervals were estimated using the DeLong method. Units: WBC × 10^3^/µL, temperature °C, IL-6 pg/mL, CD64 as X-mean (a.u.), LY% lymphocyte percentage. These multivariable models were exploratory and were used to assess incremental diagnostic information rather than to develop or validate a formal clinical prediction model.

## 4. Discussion

The present study was informed by the rationale of the work by Reed et al. [17], which examined the contribution of biomarkers to the differential diagnosis of surgical causes in girls presenting with lower abdominal pain. This is an especially challenging paediatric setting because appendicitis must be distinguished not only from nonsurgical abdominal pain but also from gynaecological emergencies, particularly ovarian/adnexal pathology. For that reason, the interpretation of any inflammatory biomarker in this population must remain clinically contextual rather than disease-specific.

In this context, the Alvarado score remains useful as a structured clinical comparator, but it cannot replace the reference standard and it should not be used to define outcome status in a diagnostic-accuracy analysis. Similarly, imaging—especially ultrasonography—remains part of routine decision-making, yet its omission from the analysed models in the present study reflects the absence of research-standardised imaging acquisition/reporting rather than irrelevance in clinical care.

IL-6 is a pleiotropic pro-inflammatory cytokine with a central role in the acute-phase inflammatory response, inducing the synthesis of acute-phase proteins such as CRP, serum amyloid A, and fibrinogen. In several clinical contexts, IL-6 increases earlier than other markers, which has led to its investigation as an early biomarker (e.g., in sepsis) [11,20]. This biological rationale supports the hypothesis that IL-6 may provide complementary information in acute appendicitis, especially at early stages or in cases with an equivocal clinical picture.

In the study cohort, 37 girls had a final diagnosis of appendicitis and 36 did not. Univariable diagnostic performance indicated that the Alvarado score had the highest discriminatory ability, consistent with its role as a structured bedside clinical tool. IL-6 demonstrated moderate discrimination, indicating that, as a standalone biomarker, it provides clinically relevant but incomplete diagnostic information. Similarly, WBC showed moderate discrimination, which is in keeping with the expected rise in leukocytosis during acute inflammation. By contrast, CRP performed less well, which is clinically plausible given that CRP may rise later than earlier inflammatory mediators. CD64 showed borderline discriminatory performance and did not add clinically useful separation in the present sample.

The limited performance of CD64 in our study warrants cautious interpretation, as this marker has been primarily established in the context of bacterial infection and sepsis. CD64 (FcγRI) is expressed on monocytes and neutrophils and increases following cellular activation, making it an attractive infection biomarker [11]. However, its clinical application involves practical challenges (method/analyser requirements, lack of fully harmonised units, potential inter-laboratory variation and cut-offs). Moreover, in a clinical scenario such as acute appendicitis, a “sepsis-like” activation pattern may not be uniformly present across cases, particularly in early or uncomplicated episodes. Therefore, the borderline performance of CD64 in this cohort does not negate its broader biological relevance, but suggests that it did not add incremental diagnostic information in this specific population and measurement setting.

In relation to the international literature, several studies have shown that IL-6 tends to rise more markedly in complicated/perforated appendicitis and may perform better when the clinical question is discrimination between complicated and uncomplicated disease [16,21,22,23]. Associations between IL-6 and other indices (CRP, neutrophil measures, total WBC) have also been reported, as well as a potential relationship with symptom duration, which is clinically plausible given the dynamic nature of the inflammatory response [13,22]. In our dataset, we did not stratify by severity/complications of appendicitis; therefore, the observed performance of IL-6 primarily reflects discrimination of “appendicitis versus non-appendicitis”, rather than severity.

This interpretation is also supported by more recent paediatric evidence focused specifically on the clinical course and severity of appendicitis. In a systematic review, Arredondo Montero et al. [13] concluded that serum IL-6 has only moderate sensitivity and specificity for the diagnosis of uncomplicated paediatric appendicitis, whereas its performance appears more favourable in complicated disease; they also noted a relationship between IL-6 levels and the duration of abdominal pain, while emphasising the need for further validation before clinically useful stratification thresholds can be defined. In a prospective validation study from the same group, IL-6 was significantly higher in children with complicated than with uncomplicated appendicitis, with median values of 60.25 pg/mL versus 17.2 pg/mL, respectively, and an AUC of 0.77 for this distinction, again suggesting that IL-6 may be more informative as a marker of severity than as a stand-alone diagnostic discriminator across all presentations [22]. In addition, postoperative data indicate that IL-6 may retain clinical relevance beyond the initial diagnostic phase: Arredondo Montero et al. [24] reported that a postoperative increase in serum IL-6 of more than 10% was associated with longer hospital stay and possible ongoing inflammatory burden, supporting the view that IL-6 reflects persistence and evolution of the inflammatory process rather than merely its onset. Taken together, these findings are consistent with our results and support a cautious interpretation of IL-6 as an adjunctive inflammatory biomarker whose clinical value may be greatest when integrated with the overall paediatric context, including disease severity, progression, and differential diagnosis, rather than when used in isolation.

A central finding of this work is that, although IL-6 has biological and clinical plausibility as a marker of acute inflammation, its addition to the Alvarado score did not demonstrate meaningful incremental diagnostic value in this sample. Practically, the Alvarado score already functions as a strong clinical classifier, leaving limited scope for measurable added value from a single biomarker. In addition, IL-6 likely overlaps with inflammatory and clinical features captured by the score, thereby reducing its independent contribution when the score is available.

At the same time, IL-6 should not be interpreted as appendicitis-specific in girls with lower abdominal pain. Gynaecological conditions—most importantly ovarian torsion or other adnexal pathology—may also be accompanied by inflammatory activation and elevated IL-6. Accordingly, the moderate performance observed here should be interpreted as discrimination within a heterogeneous emergency presentation rather than as disease-specific confirmation of appendicitis. This is precisely why biomarker findings must be integrated with clinical assessment and imaging rather than used in isolation.

Finally, several limitations should be considered. The sample size is modest for detailed subgroup analyses and clearly insufficient for formal prediction-model development; some markers had missing values; and CD64 is subject to laboratory heterogeneity in terms of standardisation and measurement platform. Operative time, length of hospital stay, and detailed treatment pathways were outside the prespecified analytical scope of this biomarker-focused diagnostic study and were not prospectively collected in a sufficiently standardized research format across all diagnostic groups to support reliable between-group statistical comparison. Imaging formed part of routine care but was not incorporated into the analysed models because it was not standardised for research purposes across all participants. In addition, the non-appendicitis group was intentionally heterogeneous, which strengthens clinical relevance but also reinforces that IL-6 is a non-specific inflammatory marker rather than an appendicitis-specific test. Despite these constraints, the study provides practice-relevant messages: the Alvarado score remains central, IL-6 may be useful as an adjunct in selected settings, and CD64 did not support clinically meaningful contribution in this cohort.

## 5. Conclusions

In conclusion, IL-6 emerged as a biomarker with moderate discriminatory ability for the diagnosis of acute appendicitis in girls presenting with lower abdominal pain, supporting its potential role as a laboratory adjunct to the initial assessment. Its performance was not sufficient for stand-alone diagnostic use, and it should not be interpreted as appendicitis-specific in a population that also includes gynaecological differentials.

In exploratory multivariable analyses, adding IL-6 to the Alvarado score did not demonstrate a clear improvement in overall diagnostic performance in this sample. Nevertheless, laboratory-based combinations including IL-6 alongside objective haematological indices achieved moderate discrimination and may be of interest when a clinical score is unavailable or cannot be reliably calculated.

Future studies should use larger prospectively assembled cohorts, prespecified analytical strategies, and explicit evaluation of clinically relevant subgroups—such as intermediate-probability presentations and complicated versus uncomplicated disease—to determine when IL-6 offers genuine incremental value and whether clinically useful thresholds can be defined.

Overall, the present findings support a cautious, clinically integrated use of IL-6 in paediatric lower abdominal pain, while reinforcing that biomarker interpretation must remain secondary to the combined clinical, laboratory, and imaging assessment.

## Figures and Tables

**Table 2:** Clinical presentation characteristics and Alvarado score at admission in girls presenting with lower abdominal pain, stratified by final diagnosis (appendicitis vs. non-appendicitis).

Variable/Category	Appendicitis (n = 37)	Non-Appendicitis (n = 36)	*p*-Value (Test)	Effect Size
Pain duration (categories) (N = 37/35)			*p* = 0.703 §	V = 0.202
–≤12 h	13 (35.1%)	14 (38.9%)		
–>12–24 h	11 (29.7%)	8 (22.2%)		
–≥7 days	2 (5.4%)	1 (2.8%)		
–>24–48 h	4 (10.8%)	7 (19.4%)		
–>48 h–≤7 days	7 (18.9%)	5 (13.9%)		
–Not recorded	0 (0.0%)	1 (2.8%)		
Temperature (N = 32/35)			*p* = 0.087 §	V = 0.334
–Afebrile/no fever	13 (35.1%)	23 (63.9%)		
–<37.5 °C	5 (13.5%)	5 (13.9%)		
–37.5–37.9 °C	6 (16.2%)	2 (5.6%)		
–≥38.0 °C	8 (21.6%)	5 (13.9%)		
–Not recorded	5 (13.5%)	1 (2.8%)		
Menarche (N = 34/31)			*p* = 0.708 §	V = 0.097
–Yes	22 (59.5%)	21 (58.3%)		
–No	12 (32.4%)	10 (27.8%)		
–Not recorded	3 (8.1%)	5 (13.9%)		
Alvarado score (N = 37/36), median (IQR)	8.00 (6.00–9.00)	4.00 (3.00–6.00)	*p* < 0.001 ‡	r = 0.770

Note: Categorical variables are presented as n (%). For categorical variables, the header row also indicates the number of available observations per group (N = appendicitis/non-appendicitis). The “*p*-value (test)” column reports the *p*-value and the test as a symbol: § chi-square test, ‡ Mann–Whitney U test. Effect sizes: V = Cramer’s V (categorical), g = Hedges’ g (*t*-test), r = rank-biserial correlation (Mann–Whitney). “Not recorded” categories indicate missing documentation for that variable.

**Table 3:** Admission haematological, biochemical, and biomarker parameters in girls presenting with lower abdominal pain, stratified by final diagnosis (appendicitis vs. non-appendicitis).

Variable	Appendicitis	Non-Appendicitis	*p*-Value (Test)	Effect Size
WBC (×10^3^/µL)	15.00 (10.10–18.30) (N = 37)	9.75 (7.08–13.83) (N = 36)	*p* = 0.002 ‡	r = 0.428
Neutrophils (NE, %)	75.35 ± 12.08 (N = 37)	68.10 ± 13.27 (N = 36)	*p* = 0.017 †	g = 0.566
Lymphocytes (LY, %)	16.67 ± 9.73 (N = 37)	21.76 ± 10.89 (N = 36)	*p* = 0.039 †	g = −0.488
Monocytes (MO, %)	7.00 (5.60–8.50) (N = 37)	7.05 (5.90–9.00) (N = 36)	*p* = 0.761 ‡	r = −0.042
Haematocrit (HCT, %)	38.94 ± 3.20 (N = 37)	38.33 ± 2.87 (N = 36)	*p* = 0.393 †	g = 0.199
Haemoglobin (HGB, g/dL)	13.00 (12.30–13.50) (N = 37)	12.70 (12.20–13.70) (N = 36)	*p* = 0.611 ‡	r = 0.070
Platelets (PLT, ×10^3^/µL)	304.00 (251.00–333.00) (N = 37)	296.50 (262.50–326.50) (N = 36)	*p* = 0.830 ‡	r = −0.030
CRP (mg/dL)	0.60 (0.10–3.60) (N = 37)	0.10 (0.03–2.42) (N = 34)	*p* = 0.167 ‡	r = 0.190
IL-6 (pg/mL)	19.41 (3.33–33.13) (N = 34)	4.10 (1.88–13.00) (N = 34)	*p* = 0.006 ‡	r = 0.391
Neutrophil CD64 (X-mean, a.u.)	6.98 (5.22–12.47) (N = 34)	6.75 (4.12–14.15) (N = 35)	*p* = 0.769 ‡	r = 0.042

Note: Continuous variables are presented as mean ± SD († Welchs’ s *t*-test; effect size g = Hedges’ g) or as median (IQR) (‡ Mann–Whitney U test; effect size r = rank-biserial correlation), according to within-group normality. N corresponds to the number of available observations per group.

## Data Availability

The original contributions presented in the study are included in the article, further inquiries can be directed to the corresponding authors.

## Abbreviations

The following abbreviations are used in this manuscript:

**Abbreviation**	**Definition**
AUC	Area under the curve
a.u.	Arbitrary units
BMI	Body mass index
BSF-2	B-cell stimulatory factor-2
CD64	Cluster of differentiation 64
CI	Confidence interval
CRP	C-reactive protein
EAES	European Association for Endoscopic Surgery
FcγRI	Fc gamma receptor I
HCT	Haematocrit
HGB	Haemoglobin
HGF	Hepatocyte stimulating factor
IFN-β2	Interferon beta-2
IL-6	Interleukin-6
IL-10	Interleukin-10
IQR	Interquartile range
log1p(IL-6)	Ln (IL-6 + 1)
LY%	Lymphocyte percentage
MO	Monocytes
NE	Neutrophils
NET	Neuroendocrine tumour
OR	Odds ratio
PAS	Pediatric Appendicitis Score
PLT	Platelets
ROC	Receiver operating characteristic
SD	Standard deviation
TNF-α	Tumour necrosis factor alpha
TNF-β	Tumour necrosis factor beta
WBC	White blood cell count
